# A Narrative Review on Multi-Domain Instrumental Approaches to Evaluate Neuromotor Function in Rehabilitation

**DOI:** 10.3390/healthcare11162282

**Published:** 2023-08-13

**Authors:** Alessandro Scano, Eleonora Guanziroli, Cristina Brambilla, Caterina Amendola, Ileana Pirovano, Giulio Gasperini, Franco Molteni, Lorenzo Spinelli, Lorenzo Molinari Tosatti, Giovanna Rizzo, Rebecca Re, Alfonso Mastropietro

**Affiliations:** 1 Institute of Intelligent Industrial Systems and Technologies for Advanced Manufacturing (STIIMA), Italian Council of National Research (CNR), Via A. Corti 12, 20133 Milan, Italy; cristina.brambilla@stiima.cnr.it (C.B.); lorenzo.molinaritosatti@stiima.cnr.it (L.M.T.); 2 Villa Beretta Rehabilitation Center, Via N. Sauro 17, 23845 Costa Masnaga, Italy; eleonora.guanziroli@gmail.com (E.G.); gasperini.giulio@virgilio.it (G.G.); franco56.molteni@gmail.com (F.M.); 3 Dipartimento di Fisica, Politecnico di Milano, Piazza Leonardo da Vinci 32, 20133 Milan, Italy; caterina.amendola@polimi.it (C.A.); rebecca.re@polimi.it (R.R.); 4 Institute of Biomedical Technologies (ITB), Italian National Research Council (CNR), Via Fratelli Cervi 93, 20054 Segrate, Italy; ileana.pirovano@itb.cnr.it (I.P.); giovanna.rizzo@itb.cnr.it (G.R.); alfonso.mastropietro@itb.cnr.it (A.M.); 5 Institute for Photonics and Nanotechnology (IFN), Italian National Research Council (CNR), Piazza Leonardo da Vinci 32, 20133 Milan, Italy; lorenzo.spinelli@polimi.it

**Keywords:** EMG, muscle synergies, EEG, NIRS, kinematics, clinical scales, biomarkers, rehabilitation, review

## Abstract

In clinical scenarios, the use of biomedical sensors, devices and multi-parameter assessments is fundamental to provide a comprehensive portrait of patients’ state, in order to adapt and personalize rehabilitation interventions and support clinical decision-making. However, there is a huge gap between the potential of the multidomain techniques available and the limited practical use that is made in the clinical scenario. This paper reviews the current state-of-the-art and provides insights into future directions of multi-domain instrumental approaches in the clinical assessment of patients involved in neuromotor rehabilitation. We also summarize the main achievements and challenges of using multi-domain approaches in the assessment of rehabilitation for various neurological disorders affecting motor functions. Our results showed that multi-domain approaches combine information and measurements from different tools and biological signals, such as kinematics, electromyography (EMG), electroencephalography (EEG), near-infrared spectroscopy (NIRS), and clinical scales, to provide a comprehensive and objective evaluation of patients’ state and recovery. This multi-domain approach permits the progress of research in clinical and rehabilitative practice and the understanding of the pathophysiological changes occurring during and after rehabilitation. We discuss the potential benefits and limitations of multi-domain approaches for clinical decision-making, personalized therapy, and prognosis. We conclude by highlighting the need for more standardized methods, validation studies, and the integration of multi-domain approaches in clinical practice and research.

## 1. Introduction

In clinical scenarios, clinical scales are the most-used tools for the evaluation of patients’ neuromotor impairment and the assessment of recovery after rehabilitation. Despite their widespread use, clinical scales present some limitations that affect the reliability, objectivity, and sensitivity as well as the feasibility of use, related to cost-effectiveness and ease of administration [1]. Besides a general consistency in repeated measurements, most clinical scales are unable to capture the entire spectrum of motor function in patients, due to limited sensitivity or ceiling and floor effects [2]. Other limitations include objectivity, inter-evaluator variability, and test responsiveness to subtle changes and the amount of time needed to apply these tests [3]. Despite being useful, clinical scales have often been proven not fully satisfactory for clinical assessments. Thus, it is crucial to identify virtuous approaches for clinical decision-making and therapy administration to provide novel insights on pathologies, as well as to minimize the long-term costs and maximize the recovery of rehabilitation procedures. One of the most promising approaches is the use of multi-modal devices and multi-domain analysis techniques, which allow for the detection of novel relationships between variables and the acquisition of new knowledge on physiological processes.

Therefore, in recent decades, clinicians have started to rely on the availability of tools, devices, and techniques for the analysis of biological signals, exploiting their added value in comparison to the standard clinical practice; the innovative, highly advanced, and pioneering approaches favor the adoption of multi-modal solutions and the improvement of the assessment of rehabilitation effectiveness in a plethora of pathologies. This process boosts the progress of research in clinical and rehabilitative practice and improves the understanding of the pathophysiological changes occurring during and after rehabilitation, overcoming the low sensitivity of traditional clinical practice. Instrumental measurements, such as kinematic recordings, electromyography (EMG), electroencephalography (EEG), or near-infrared spectroscopy (NIRS), are widely used in clinical scenarios and can provide quantitative assessments of the patient’s health state. Each instrumental assessment explores and provides information about a specific domain: kinematic recordings allow us to quantify motor performance and quality of movement by directly assessing the motor outputs; EMG measures the activity of muscles and can be useful for muscle-fatigue detection, force estimation, and motor-control analysis; EEG measures the electrical activity of the brain and can monitor the complex neuronal activity and its changes; NIRS measures the hemoglobin concentration in human tissue and can assess the hemodynamics in the brain cortex or muscles. However, since motor and neural functioning are complex structures involving individual, environmental, and contextual factors, a multi-modal comprehensive characterization of the patient is needed to overcome the limited view that is provided when using only one sensor at a time. It is unlikely that a single outcome measure may adequately assess the degree of neuromotor functions and differentiate between key elements of the same function. Indeed, multi-domain approaches combine information and measurements that are collected with sensors from different domains, such as kinematics, EMG, EEG, NIRS, and others. Each domain gives specific information that, combined with the other measurements, may provide a more comprehensive understanding of the state of the patient. This may explain why, in research studies, different clinical outcome measures have been integrated to capture different domains of the same function and different domains of the International Classification of Functioning, Disability, and Health (ICF) (i.e., impairment vs. activity vs. participation) to gain a more complete picture of functioning. In fact, in many neurological disorders, the impairment regards the neural and sensorimotor systems at different levels, and, therefore, a detailed quantitative assessment is essential for the definition of rehabilitation treatment and the evaluation of the recovery [4]. Therefore, clinical interventions have to be tailored to the individual in order to evaluate the recovery. Multi-modal and multi-parameter assessments provide information on different domains that can be used to identify the specific patterns of the functional organization of the neuromotor control of the patient and can help to address the rehabilitation [5].

Multi-modal approaches allow us to choose the most effective treatments and, prospectively, provide the best care methods for patients. These methodologies may be integrated into clinical practice to support clinical decisions, to provide a comprehensive assessment of the patient and quantitatively evaluate motor recovery during rehabilitation. However, the main findings achieved using multi-parameter assessments and the potentiality of combining measures from multiple domains have not been summarized to guide future directions in the clinical field.

This paper provides a detailed overview of the current scientific research based on multi-modal and multi-parameter assessments in the clinical field and suggests how to improve scientific and clinical research to promote the use of multi-domain approaches, in order to foster the application of multi-parameter assessment in clinical practice by showing clear evidence of its potential. This overview focused mainly on musculoskeletal and central nervous system (CNS) assessment in rehabilitation.

## 2. Methods: Rationale and Research Questions

This narrative review aims at collecting and reviewing the main multi-domain instrumental approaches that are currently employed in clinical assessments, with a particular focus on the rehabilitation field. We would like to (i) provide a summary of the main domains assessed with instrumental technologies, (ii) summarize which combinations of multi-domain instrumental approaches have been used so far, (iii) describe the main achievements obtained, and (iv) critically comment on the future directions for the field.

The findings that will be summarized come from a vast field and from variable approaches. A screening of the literature was performed, and the most representative articles were selected based on the authors’ experience. Only papers available in the main databases (Web of Science, PubMed, Scopus, and Google Scholar) and written in English were considered for selection. From this analysis, the main advantages and disadvantages of the multi-domain approaches will be discussed, highlighting how future research and clinical practice would be impacted by these techniques.

## 3. Results

### 3.1. Summary of the Main Domains of Assessment and Main Achievements

**Figure 1:**
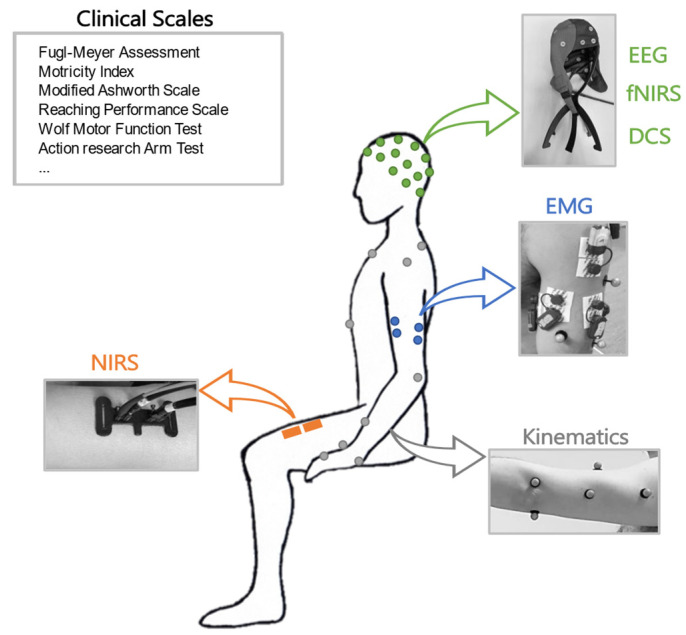
Schematic representation of clinical assessments in different domains: clinical scales, EEG, fNIRS, DCS, EMG, kinematics, and NIRS.

A scheme of the main instrumental approaches related to different physiological domains investigated in this work is shown in Figure 1.

#### 3.1.1. Clinical Scales

People with neurological conditions can suffer from several impairments, ranging from paresis associated with decreased strength and sensation to increased muscle tone, loss of inter-joint coordination, and impaired control of voluntary movement that results in slow, imprecise, and uncoordinated task-related movement [6]. These impairments often cause limitations in activities of daily living and may decrease the quality of life. Although many studies have investigated the efficacy of various rehabilitation interventions, how to improve functions after CNS damage remains an important challenge [7]. Clinical outcome measures are essential for improving clinical practice and for evaluating the efficacy of rehabilitation interventions.

Several systematic reviews, guidelines, and consensus-based recommendations have evaluated the psychometric properties and/or feasibility of clinical outcome measures [8] in neurologic conditions. Other reviews described the different ICF domains (namely body function, activities, and participation) covered by each measure [9] and provided an overview of the frequency of use in clinical practice and research [1]. 

Within the ICF Body Structure/Body Function level, a variety of assessment scales exist to evaluate motor impairment after stroke, designed to capture aspects such as joint range of motion (ROM), strength, synergistic execution of movements, gross motor capabilities, and object manipulation. These assessments concern movement strategies used to accomplish a task and try to identify the factors that may affect the performance of a task. In the last few decades, the focus of rehabilitation has slowly shifted from ICF function level towards activity and participation level [10]. The limitations at the activity level are associated with limitations in the execution of activities of daily living, resulting in greater dependency, restricted social participation, and a decreased quality of life experienced by those with neurological conditions. However, the relationship between function/impairment level and activity level (and participation as well) is still poorly understood [11]. Information about activity level, although very useful in clinical settings, may not reveal valid information about the functioning of a patient in daily life.

In the last few years, several recommendations combining existing evidence, clinical practice guidelines, and expert consensus have provided a core set of outcome measures for upper- and lower-limb assessment in neurological rehabilitation [12]. Most of these core recommendations have prompted the usage of the Fugl–Meyer Test (FMT) for upper- and lower-limb-impairment assessment [13] to assess sensory and motor function, balance, and coordination. At the activity level, the Action Research Arm Test (ARAT) [14] and the Wolf Motor Function Test (WMFT) [15] measure upper-limb impairments and activity limitations. The widespread adoption of core assessments could improve the quality of clinical practice and the effectiveness of interventions.

Another drawback of the clinical outcome measures is that they are insufficiently sensitive to capture the quality of sensorimotor performance. This impedes the ability to clearly distinguish behavioral restitution or compensation, which is essential for understanding the neurological mechanism of sensorimotor recovery post-stroke [16]. 

A better understanding of the relationship between movement quality and the ability to perform functional activities can be achieved with a detailed analysis of the joints/body segments involved in accomplishing a particular task [6]. This is usually not part of standardized clinical outcome measures since they mainly focus on task accomplishment. Only a few outcome measures are focused on the assessment of quality aspects of functional movements [17]. Technologies such as wearable sensors, robots, force sensors, and 3D motion-capture techniques allowing the objective measurement of movement kinematics and kinetics were suggested as the best way to tackle this problem [18]. 

Clinical outcome measures have also been used as predictors of recovery potential [19]. A range of models to predict upper- and lower-limb motor outcomes have been published. Measurable grip strength at 1 month or the presence of shoulder abduction or finger extension at 3 days post-stroke are suggested as plausible predictors for upper-limb recovery. Prediction models only including clinical assessments have shown to be inferior compared to the models combining clinical and neurophysiological or neuroimaging techniques. This seems to be particularly valid for patients with initial poor motor function [20].

#### 3.1.2. Kinematics

**Figure 2:**
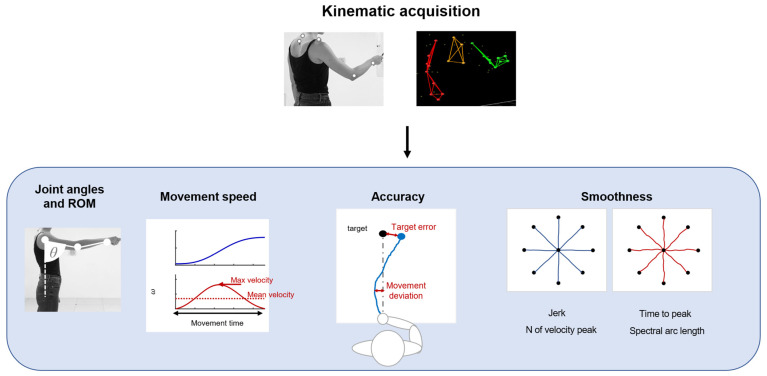
A schematic representation of the application of kinematics, such as joint angle and ROM analysis. Parameters based on movement speed, to evaluate movement accuracy and smoothness.

Kinematic evaluations can provide quantitative and objective information about motor output associated with tasks and allow for monitoring the administration of therapeutic techniques [21]. Kinematics measures refer principally to the execution of a movement and its repeatability, such as articular angles, range of motion (ROM), and measures derived from the end-effector movement. A summary of common applications is shown in Figure 2.

These parameters can be measured with optoelectronic systems that require a set of infrared cameras and retroreflective markers placed on anatomical landmarks of the patients, or marker-less systems, such as RGB-depth cameras or IMUs. Parameters related to movement speed are usually movement time, mean velocity [22], and peak velocity [23]. In upper-limb applications, the accuracy of movement can be evaluated with metrics derived from the end-effector movement: movement deviation from a theoretical or desired trajectory [24] and target error, measured as the end-point error around the target placement [25]. These measures are often employed during robotic rehabilitation, but they can be used also in free body movements. Kinematics also provides information about the fluidity and repeatability of movements. In particular, measures of movement smoothness are usually considered for the evaluation of motor-control improvement and dexterity. These measures are based on the velocity profile or jerk, which is the third time derivative of the position. Smoothness metrics based on speed usually employed in clinical scenarios are the number of velocity peaks [26], the ratio between the mean speed and the peak speed [27], time to peak velocity [28], and spectral arc length [29]. Other measures are based on jerks, such as the normalized jerk [22,28].

Kinematic measures provide insight into the movement execution and functional outcomes of the neurological system performance of the patient; however, they cannot identify coordination patterns and the underlying mechanisms of impairments, which can be detected with other neurophysiological measures [30].

#### 3.1.3. EMG

**Figure 3:**
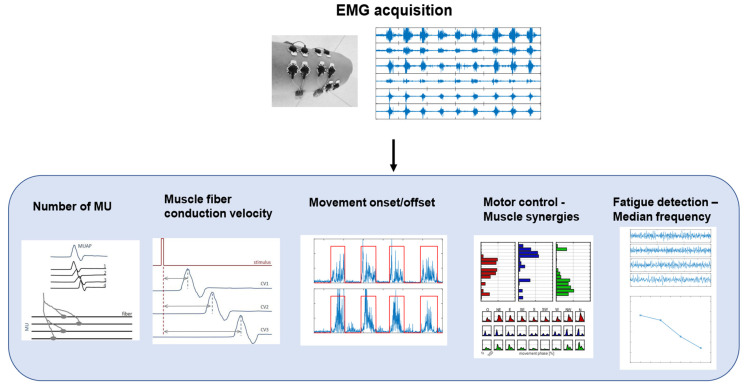
A schematic representation of EMG signal processing workflow and biomarkers. The figure shows different applications of EMG signals in clinical and motor rehabilitation, such as estimation of number of motor units (MU), computation of muscle-fiber conduction velocity, detection of movement onset/offset, analysis of motor control with muscle synergies, and detection of muscle fatigue with median frequency.

Surface EMG is a non-invasive technique used to record the activity of the muscles under the electrodes placed on the skin [31]. The acquired signal is the train of the motor-unit action potentials (MUAPs) generated by the muscle fibers of the motor unit, in response to nervous stimulation [32]. An EMG signal can be analyzed in both time and frequency domains, and some common applications are shown in Figure 3.

Common applications in the time domain are the estimation of the number of motor units (MUs), the detection of muscle activity, and the analysis of muscle co-contraction. The number of MUs has been identified as a biomarker for the progression of neurodegenerative diseases, such as amyotrophic lateral sclerosis (ALS) [33]. The detection of muscle activity and, specifically, the identification of the onset and the offset of the activity, is useful in clinical practice for motor-control analysis or for the evaluation of the results of interventions, such as orthopedic surgeries [34]. EMG is also at the basis of motor-control theories based mainly on time and spatial domain analysis, such as muscle synergies, which provide insight into the control mechanisms for motor planning [35]. In particular, muscle activation patterns can be decomposed into a limited number of modules, called synergies [36], which provide insight into the organization of muscle activation during motor tasks. According to this theory, muscle co-activation patterns are extracted using decomposition algorithms to obtain spatial and temporal components of muscle activations, which, respectively, could be interpreted as co-activating muscles and their time recruitment. This analysis can provide information on muscular coordination and motor control [37]. The analysis of EMG signals in the frequency domain is usually related to the detection of muscular fatigue. Muscle fatigue is the result of a complex mechanism that causes structural and energetic changes in muscles and, therefore, it can be detected with EMG signals [38]. In particular, the median frequency (MF) and mean power frequency (MPF) of EMG signals show a decreasing tendency during fatigue [39] and, therefore, they can be used as biomarkers for fatigue [40], preventing patients, athletes, or workers from injuries.

Despite its numerous applications, the EMG signal is affected by the structure and the placement of the electrodes [41] and by the number and properties of active fibers, such as type and conduction velocity [42]. Another phenomenon affecting the EMG signal is cross-talk, which is the recording of electric signals from nearby muscles that are not investigated and that overlap with the signal from the muscle under investigation [43]. Despite the possible presence of these noises, surface EMG is a useful and powerful tool that provides insight into human physiology and supports the diagnosis of neuromuscular diseases. Indeed, EMG signals are used in a wide variety of applications in both research and the clinical field, such as muscle-fatigue detection, force estimation, and motor-control analysis [44].

##### Applications of EMG in Rehabilitation

In clinical scenarios, EMG analysis has been used for a variety of assessments. Current applications of EMG are mainly related to physiological investigation, the monitoring of neurological disorders, and the planning of treatments [44]. The evaluation of muscle activity and coordination is a useful tool for the assessment of motor impairment and for targeting the rehabilitation activity in patients with neuromotor disorders, such as post-stroke patients, since they usually show abnormal muscular activity, including spasticity, weakness, or spatiotemporal patterns [45]. Motor control theories can be used for the quantification of motor-control abnormalities [46] and changes in muscular activation patterns [47]. In recent years, interesting applications of EMG were found in prosthetic control [48], in which amputees can perform simple yet useful movements by controlling automatized prostheses with residual EMG near the amputated region. Several EMG pattern-recognition algorithms are implemented for myoelectric prosthesis control [49]. EMG signals in clinical practice have also been employed for characterizing the impact of motor abnormalities on motion by identifying changes in EMG median frequency at fatigue [50]. EMG signals were also used to evaluate the effects of rehabilitation on muscle fatigue and force capacity in inpatients, showing that force capacity increased without increasing fatigue [51].

#### 3.1.4. EEG

EEG is a technique that measures the electrical activity of the brain from electrodes attached to the scalp. EEG has a high temporal resolution, meaning that it can capture fast changes in brain activity with millisecond accuracy [52]. EEG signals reflect the synchronous activity of large populations of neurons, and they are characterized by different frequency bands that correspond to different physiological and behavioral conditions. For example, delta waves (0.5–4 Hz) are dominant during deep sleep, theta waves (4–8 Hz) are related to memory and emotion, alpha waves (8–13 Hz) indicate relaxed wakefulness, beta waves (13–30 Hz) reflect alertness and concentration, and gamma waves (30–150 Hz) are associated with cognitive processing and sensory integration [53]. 

With the advent of high-density systems, it is possible to monitor whole-brain neuronal activity with a better spatial resolution, which is more suitable for exploring functional network organization [54]. Indeed, EEG can also show how different brain regions communicate with each other, by measuring the functional or effective connectivity between them. Functional connectivity (FC) reflects the statistical dependence or correlation between two signals, while effective connectivity (EC) reflects the causal influence or direction of information flow between them [55]. EEG connectivity can be estimated with different metrics, such as coherence, phase synchronization, Granger causality, or transfer entropy [56,57], both in the time and frequency domain. 

Moreover, graph analysis has been successfully employed to concisely describe the brain network’s integration and segregation behavior in communication [58]. The brain is thus described as a complex network, where specific cortex areas represent nodes and the links between these nodes represent the functional interaction between these cerebral regions. It has been found that the human brain exhibits a small-world behavior—a balance between a local and global integration of networks.

**Figure 4:**
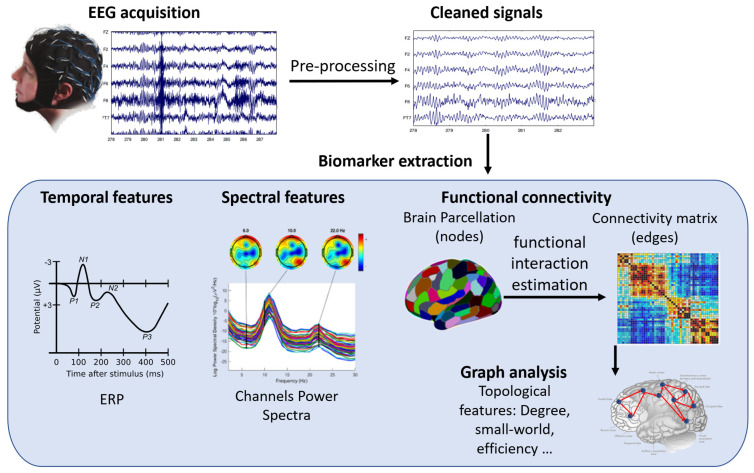
A schematic representation of EEG signal processing workflow and biomarkers. The figure shows the steps involved in acquiring, preprocessing, analyzing, and interpreting EEG signals for different applications in motor rehabilitation. The biomarkers include event-related potentials (ERP), power spectrum, functional connectivity, and graph analysis.

EEG is thus a valuable tool for studying how the brain changes and recovers its functions after injury or disease, such as stroke, Parkinson’s disease (PD), cerebral palsy (CP), spinal cord injury (SCI), or traumatic brain injury (TBI). EEG has several advantages over other neuroimaging techniques, such as being affordable, portable, easy to use, and adaptable to different situations. EEG can work both in rest and movement conditions, and it can be combined with other modalities, such as EMG, kinematics, functional NIRS (fNIRS), or transcranial magnetic stimulation (TMS). EEG can reveal various aspects of brain function that are relevant for motor rehabilitation, such as event-related potentials (ERPs), power spectra, and connectivity measures. ERPs are time-locked changes in EEG signals that reflect the brain’s response to specific stimuli or events. Power spectra show the distribution of EEG signal energy across different frequency bands. Connectivity measures show how different brain regions interact with each other. Figure 4 shows how EEG signals are processed and what kind of information can be obtained from them.

##### Applications of EEG in Rehabilitation

EEG is a widely used technique to study the brain changes that occur during and after post-stroke rehabilitation, across different stages of recovery [59]. EEG biomarkers, such as the ratio of slow (delta/theta) to fast (alpha/beta) waves, can predict motor outcomes in stroke patients, as they indicate the level of arousal and alertness of the brain [60,61]. EEG can also investigate other neurological disorders that affect motor function, such as PD, which is characterized by reduced motor-evoked potentials and altered EEG microstates [62]; CP, which shows abnormal EEG patterns and connectivity [63,64]; SCI, which affects the cortico-spinal communication and motor control [65]; and TBI, which disrupts the functional network organization and integration [66,67].

In addition to traditional biomarkers, more recently, FC investigation proved to be particularly interesting in the study of rehabilitation effects in those pathologies derived from the disruption of information transfer between brain regions and helped to explore how induced brain plasticity may play an important role in functionality recovery [68].

In the following text, we focus on summarizing more recent findings of FC analysis, undirected or directed, in the field of motor rehabilitation treatments.

Brain connectivity in the resting state (RS) is one of the most investigated conditions since it is the easiest experimental protocol that can be performed with patients of all grades of impairments, and it has been demonstrated that RS connectivity is predictive of motor-function recovery in stroke patients [69]. Nevertheless, studies have also been conducted during the proper execution of tasks [70,71] or motor imagery [72] protocols to investigate movement-related network configuration.

Most of the studies evaluating the effect of rehabilitation in motor recovery focused their analysis on motor-network characterization. However, altered motor-network FC has also been found with higher-order cognitive control networks such as default mode networks, executive control networks, and dorsal attention networks. Therefore, connectivity patterns have been recently investigated both within and between RS large-scale networks [73,74,75].

In the literature, the major results of connectivity analysis in the rehabilitation field are focused on stroke recovery, comparing stroke patients with control groups [69,76,77,78] and evaluating the effect of different rehabilitation treatments [79,80,81]. In most works, an altered inter-hemispheric connectivity pattern was found. Homologous regions of the two hemispheres show reduced connectivity in the acute stage, which gradually returns to a normal level in sub-acute and chronic stages both during rest and motor execution [70,82,83]. Indeed, brain network reorganization has been demonstrated to depend on time after stroke [84], and an increase in inter-hemispheric connectivity, particularly between the primary motor cortexes in alpha and beta frequencies, was found to positively correlate with motor outcome improvement [71,82,83]. Conversely, an increase in RS-directed connectivity measures, from pre-motor towards primary motor intra-hemispheric regions, was found in sub-acute patients assessed before and after rehabilitation treatment [85]. Hoshino et al. in 2021 found higher intra-hemispheric FC in both hemispheres in RS and during ankle movement [71]. Wu et al. in 2015 found a positive correlation between motor outcome and an increase in coherence between ipsilesional pre-motor and primary motor cortexes in chronic patients after one month of rehabilitation [86]. This alteration in the communication may be due to alterations between the segregation and integration of information between affected and non-affected hemispheres [68].

Philips et al. in 2017 proved that topographical measures of integration and segregation among functional networks may be useful biomarkers of post-stroke motor recovery, suggesting their employment for the prognosis and evaluation of therapeutic outcomes [87]. Many studies employed graph analysis [88], reporting that small-worldness reduces in stroke patients when compared to healthy subjects [76,77]. Small-worldness in RS brain networks was also suggested to represent a biomarker of functional recovery in stroke patients since a correlation with motor outcome was found [77]. Molteni et al. found an increase in the node strength of the contralesional primary motor cortex and ipsilesional pre-frontal cortex after exoskeleton training in subjects with lesions in the non-dominant hemisphere, as well as a restoration of the interactions between primary motor and premotor cortexes after rehabilitation [80].

As for large-scale intra-network connectivity, Romeo et al. investigated the interaction of 14 RS networks and their correlation to different impairment domains in a cohort of 30 sub-acute/chronic stroke patients. Interestingly they found a correlation between dorsal attention networks and language network FC with motor indexes [75]. Wang et al. in 2018 explored neurological changes after guided or non-guided robot hand training in 24 chronic stroke patients. They found that only the robot-assisted group showed motor improvement and found an increase in the temporal variability of six large-scale networks, including somatomotor, attention, auditory, and default mode networks [73]. 

#### 3.1.5. NIRS

NIRS is a non-invasive optical technique that allows for the measurements of hemoglobin concentration in human tissues using light in the near-infrared region (650–900 nm). Light is shone into the biological tissue and recollected from the same by means of optical fibers. The injected photons travel in the whole volume under the optical fibers and the features of the recollected signal depend on the different tissue optical properties, i.e., the absorption and diffuse coefficients [89].

The main physiological parameters investigated with this technique are the oxygenated-(O_2_Hb) and deoxygenated-(HHb) hemoglobin concentrations, allowing for the calculation of tissue total hemoglobin (tHb) content and tissue oxygen saturation (SO_2_ = O_2_Hb/tHb). 

Among the applications of NIRS, we mention the measure of the cortical response function (i.e., an increase in O_2_Hb and a concomitant decrease in HHb in the brain cortex) after the administration of stimuli of different natures. In this case, the optical probe is placed on the scalp and photons travel through the head, reaching the brain cortex after going through the skin, the skull, and the cerebrospinal fluid. This kind of study is referred to as functional NIRS (fNIRS) [89]. Another important application of NIRS encompasses the assessment of the muscle oxidative metabolism through the measurement of skeletal muscle fractional O_2_ extraction, typically represented by a concomitant increase in HHb and a decrease in O_2_Hb [90]. In this case, the probe is placed on the limb and photons travel through superficial layers, such as skin and fat, before reaching the muscular tissue. 

Because of the hardware simplicity and moderate price, the most common approach for NIRS acquisitions is based on the employment of continuous wave (CW) optical radiation. CW NIRS allows us to retrieve relative variations of the hemodynamic parameters with respect to an arbitral baseline [91]. In this case, the sensitivity to deeper tissues is obtained by increasing the source–detector distance or employing more source–detector pairs. On the contrary, by adopting a time-domain (TD) approach to NIRS, it is possible to retrieve the absolute values for the hemodynamic parameters and to better discriminate the investigated tissues in depth. This is obtained, however, with more complex instrumentation [92]. In the literature, we also find devices based on the frequency-domain regime (FD).

**Figure 5:**
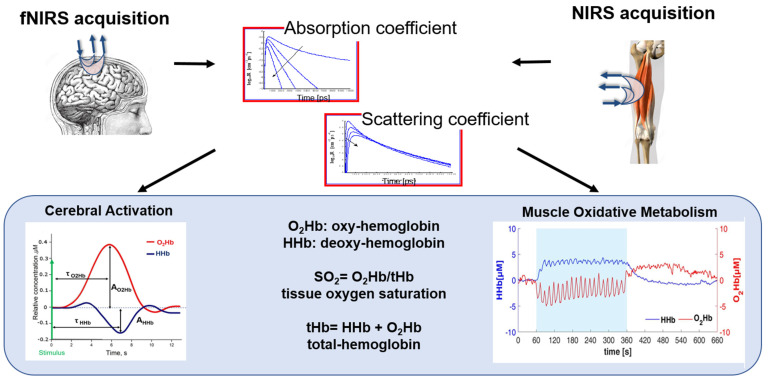
A schematic representation of fNIRS and NIRS signal processing workflow and biomarkers. The figure shows the steps involved in acquiring, preprocessing, analyzing, and interpreting NIRS signals for different applications in motor rehabilitation. The biomarkers include the hemodynamics parameters, i.e., oxygenated-(O_2_Hb), deoxygenated-(HHb), and total-(tHb) hemoglobin, and the tissue oxygen saturation (SO_2_).

Finally, NIRS is a relatively low-cost, noninvasive technique that can be made multichannel and can be easily applied at the bedside for the continuous monitoring of the hemodynamic parameters. Moreover, it is compatible with various other clinical techniques. For these reasons, it has already been employed in different scenarios and different clinical settings, even if it can be still considered quite a new technology. In the following, some examples are presented, giving wider importance to the rehabilitation scenario and focusing on both the brain and muscle applications. In Figure 5, a schematic workflow and extrapolated biomarkers for NIRS and fNIRS are shown. 

Lastly, another optical technique was recently introduced in combination with fNIRS: diffuse correlation spectroscopy (DCS), which allows for the investigation of the motion of red blood cells and calculating cerebral blood flow, exploiting the coherence loss of highly coherent laser light when it diffuses in biological tissues [93]. For further details and applications, see the following sections. 

##### Applications of NIRS in Rehabilitation (Brain)

One of the main applications of fNIRS is the measure of brain cortex hemodynamics in response to neural activity, which allows researchers to investigate a wide range of cognitive and motor processes connected to rehabilitation. It has also been widely leveraged in clinics to assess cerebral hemodynamics in patients suffering from stroke [94], TBI [95], or dementia [96]. Recent technological improvements allowed also its exploitation in delicate environments such as neonatal intensive care units (ICUs), where fNIRS is particularly suitable for assessing the cerebral autoregulation of preterm infants [97]. 

In the neuro-rehabilitation contest, compared to other neuro-imaging techniques such as fMRI, whose studies in this framework are limited to the upper-extremity exercises, fNIRS allows us also to investigate brain oxygenation during the exercise of the lower limbs, being less prone to motion artifacts. An important type of study where fNIRS is exploited concerns the monitoring of the rehabilitation process, to investigate the mechanisms of functional recovery after brain injury. In other cases, fNIRS was used as a therapeutic tool to develop brain–computer interface modules (BCI) [98]. Many papers have been published exploiting fNIRS as a diagnostic tool, and most of the published studies are focused on stroke survivors and patients with cerebral palsy [99]. In the past few years, great attention has been paid to investigating human–robot interaction, studying the hemodynamic response to robot-assisted motor tasks. In 2022, Bonnal et al. studied the cerebral activations during walking with an exoskeleton at different levels of assistance, and they observed that the amplitude of the activations was strictly related to the level of effort during gait [100].

Due to fNIRS acquisitions on the cerebral cortex, we have information about neurovascular coupling. This process is always more considered, together with other parameters acquired in the clinical practice, in order to have a comprehensive view of the cerebral cortex’s functioning and state. With fNIRS, it is easier to perform experiments when the subjects are walking or performing rehabilitation exercises, thanks to the possibility of making this technique portable [101,102]. Recently, it was also demonstrated that a major comprehension of the fNIRS signal could be possible with the employment of concomitant physiological signals (such as arterial partial pressure of CO_2_, blood pressure, skin conductance, skin temperature, heart rate, respiratory rate, photoplethysmography, and others), which can be also used for regressing out physiological confounding components in fNIRS signals [103]. In the future, fNIRS instrumentation will increasingly be integrated with these and other emerging modalities, such as eye-tracking and augmented and virtual reality devices.

##### Applications of NIRS in Rehabilitation (Skeletal Muscle)

The employment of NIRS techniques on muscular tissue allows for the determination of local musculoskeletal O_2_ saturation and oxygen consumption ($\dot{V}$O_2_) as a measure of muscular oxidative metabolism [104]. In a recent review from Tuesta et al., an overview of the effects on muscular oxygenation of the exercises in a clinical scenario is presented [105]. In particular, different pathologies were considered, such as multiple sclerosis, orthopedic disorders, acute myocardial infarction, heart failure, type 2 diabetes mellitus, chronic kidney disease, metabolic muscle, and peripheral artery diseases. Concerning the rehabilitation field, fewer examples can be found: the evolution of functional impairments and the rehabilitative intervention were monitored [90], and the loss of muscle oxidative capacity associated with aging or diseases was evaluated [106]. Skeletal muscles were also monitored during early rehabilitation after heart failure, which cause a disturbance of the peripheral perfusion, to target therapeutic strategies [107]. Manfredini et al. identified novel muscle metabolism biomarkers to evaluate muscle adaptations in patients with peripheral artery disease [108]. The effect of the rehabilitation was also evaluated in patients with coronary artery diseases combining NIRS acquisitions and vascular occlusion tests [109]. Finally, muscular metabolism was also assessed during post-stroke rehabilitation [110].

### 3.2. Summary and Achievements of the Main Multi-Domain Instrumental Approaches

In this section, we reported multi-domain approaches that were found to be most diffused in clinical applications. We discuss in detail the combinations of instrumental approaches from different domains.

#### 3.2.1. EEG + EMG

EEG and EMG signals regard functional activities that are strictly correlated: the EEG measures the activity at the brain motor cortex, where the motor commands start, while the EMG quantifies the muscle activations that are generated by those commands. Therefore, EEG + EMG analysis may give insights into how each of the two domains is affected by the impairment and restored with the rehabilitation, supporting the therapy decision process and the evaluation of the motor recovery [111]. The most diffused application of the combined EMG and EEG signals is the analysis of cortico-muscular coherence (CMC), which assesses the functional connections between the brain motor cortex and the associated muscles [112]. CMC can assess functional changes between acute and chronic stages in post-stroke patients [113] and evaluate the positive effects of rehabilitation on motor recovery [114]. The analysis of EEG combined with EMG was employed for the evaluation of exoskeletons [115] or robotic devices [116] used for rehabilitation, finding that both brain connectivity and muscle activations improved with therapy. Relations between cortical activity and muscular activations have also been investigated with mutual information for the detection of movement intention, which is needed to increase the effectiveness of the rehabilitation of post-stroke patients [117]. Recent applications of combined EEG and EMG approaches also include a measure of directed information flow that has been adopted to investigate the effects of electrical stimulation during cycling on neuro-muscular coupling in chronic stroke [118] and the fusion of EEG and EMG signals for device-guided therapy [119].

#### 3.2.2. Kinematics + EMG

Biomechanical and neural activity provide complementary information about the neuro-musculoskeletal system of the patient with an objective approach [30]. The combined analysis of kinematics and electrophysiological measurements gives a complete characterization of the sensorimotor control, highlights differences between pathologic and normal subjects, and monitors the ongoing development of motor recovery [120,121]. Kinematics provides information about the motor output, while EMG signals give insights into the neurophysiological mechanisms that generate the movement. The two assessments may provide not overlapping information, as the same movement may be generated with different combinations of the redundant muscle system. Typical examples of kinematic analysis combined with EMG measures allowed us to examine the effects of rehabilitation in hemiplegic children [122], revealing that the combined analysis can highlight the differences in the postural responses between the less-affected and more-affected sides. In patients with spasticity, biomarkers of spasticity were extracted from kinematics and EMG describing the biomechanical and neurogenic components of spasticity [123]. The combined use of kinematics and EMG allowed us to evaluate their motor condition, discriminating the causes of spasticity that could not be found with traditional clinical assessment and targeting rehabilitation [124]. A novel approach for fusing both domains is the mixed-matrix factorization (MMF) algorithm that allows for extracting both muscular and kinematic synergies that can give insights into motor control by linking the muscle and task spaces in the same factorization [125].

#### 3.2.3. Kinematics + EEG

EEG measures can provide complementary information on motor cortex activations and brain connectivity that can be related to the motor output evaluated by kinematic measurements. In post-stroke patients, both domains were positively affected during rehabilitation [126], indicating that neuromotor recovery reflects in movement performance and neural activations coherently in multi-modal assessments [127]. Neuromotor biomarkers for the evaluation of patient performance were defined by relating kinematics to EEG signals, which could describe both the cognitive and motor state of the patient that has to learn to interact with the environment [128]. During robotic-assisted therapy, EEG showed that neuroplasticity was stimulated and was related to an improved motor outcome [129]. EEG and kinematics were also assessed together with EMG for the evaluation of the effects of rehabilitation on post-stroke patients [130,131]. In particular, Belfatto et al. [130] showed that improvements found in one of the domains could not be spotted in other domains and that multi-parameter approaches can detect finer improvements that could not be identified by clinical scales only. Pierella et al. [131], instead, integrated the information from the different domains, finding that clinical improvements correlated with changes in kinematics, muscle synergies, and spinal maps after rehabilitation in stroke patients.

#### 3.2.4. NIRS + EMG

The combination of EMG with NIRS allowed us to assess muscle characteristics from both an electrical and hemodynamic point of view [132]. Principal applications regard the characterization of muscle fatigue in healthy subjects [133,134,135], showing that spectral EMG features are related to hemodynamic parameters and provide complementary information on muscle fatigue. Both domains were analyzed for assessing patients with lower-back pain, showing that pain was not related to impaired muscle fatigability or oxygen consumption [136], or muscular pain [137], finding that physical exercise stimulates oxygenation and may have beneficial effects on muscular pain. Very few studies used these techniques for evaluating the effects of rehabilitation in chronic stroke patients [138] and those with spinal-cord injury [139], showing that rehabilitation stimulates not only muscular activity but also tissue oxygenation. The effect of the recruitment pattern of muscular fibers on muscular oxygenation was described for patients with incomplete spinal cord injury, where also the impact on their rehabilitation process is discussed [140].

#### 3.2.5. fNIRS + EEG

The multi-modal approach EEG-fNIRS provides complementary information to explore neurovascular coupling. In the literature, it is possible to find tens of applications of combined fNIRS-EEG acquisitions, because of the orthogonality of the neurophysiological information provided by the two technologies. Among the clinical applications, rehabilitation is present for only 8% [141], probably because of the complexity of the acquisition set-up for the EEG and its sensitivity to motion artifacts. Their combined use is of particular interest for brain connectivity investigation [142]. Recently, it has been used to assess post-stroke cortical reorganization and identify biomarkers of motor recovery after 4 weeks of rehabilitation [143]. A hybrid EEG-NIRS device combined with body motion capture allowed us to distinguish PD with more than 83% accuracy for each individual [144]. Moreover, hybrid EEG-fNIRS systems have been used to assess cortical connectivity in stroke rehabilitation with transcranial direct current stimulation [145,146], and more recently to monitor non-responding patients with acute brain injury, obtaining 99% accuracy in distinguishing patients that subsequently failed to recover consciousness [147]. The fusion of EEG and fNIRS also provides a useful approach to evaluate guided robot-assisted rehabilitation [148]. For example, Wang et al. found that BCI-based neurofeedback training in chronic stroke subjects increased their EEG event-related synchronization/desynchronization during motor imagery and enhanced cortical activity measured with fNIRS [149].

#### 3.2.6. fNIRS and NIRS + DCS

Combining tissue oxygenation (measured by NIRS), blood flow (by DCS), and arterial saturation (estimated by a commercial pulse oximeter) it is possible to measure the tissue metabolic rate of oxygen consumption. In the literature, we can find examples of their combined clinical application on the neonatal brain. In 2010, Durduran et al. measured cerebral hemodynamics and oxygen metabolism in neonates with congenital heart defects, and they observed a good agreement with gold-standard arterial spin-labeled magnetic resonance imaging [150]. More recently, Rajaram et al. exploited a hybrid broad-band fNIRS and DCS device (NNeMo) to study cerebral hemodynamic and metabolic changes of neonates in ICU during ventricular tap [151]. De Carli et al. tested a hybrid TD fNIRS and DCS device to assess cerebral changes in postnatal transition [152]. No contributions to the neurological rehabilitation field were found.

Recently, the DCS technique was also applied to periphery body compartments to obtain information about systemic tissue perfusion and vascular diseases [153]. Many papers have been published on the combined NIRS and DCS application on muscle, with the aim of the assessment of both muscular perfusion and metabolism. Zanoletti et al. developed a hybrid TD NIRS and DCS device for evaluating endothelial function and metabolism for patients in the intensive care unit (ICU) and preventing extubation failure during the weaning from mechanical ventilation [154]. Baker et al. studied the effects of supervised exercises in patients with peripheral artery disease, observing that supervised exercise training improved patient ability to increase microvascular calf-muscle blood flow and oxygen extraction during physical activity [155]. In 2019, dynamic NIRS and DCS measurements were performed during cycling by Quaresima et al., successfully testing an algorithm to remove movement artifacts, paving the way for the exploitation of hybrid NIRS-DCS devices in rehabilitation [156].

#### 3.2.7. NIRS + Others

A multi-domain assessment could also be useful in muscular applications. In particular, there are many examples where both the central and peripheral aerobic functions have to be monitored, such as in chronic heart failure (CHF) patients [157]. To better understand the impact of exercise training, pulmonary ventilation and cardiac output measurements should also be acquired by a computerized metabolic cart [158]. The assessment of the muscle-fiber recruitment patterns on muscle oxygen utilization is of interest to gain a comprehensive knowledge of the muscular activation patterns. To this purpose, a combined acquisition with EMG is needed, which can also be useful to assess local muscular fatigue.

## 4. Discussion

### 4.1. Advantages of Multi-Domain Approaches

Instrumental assessments provide quantitative information on the neuromotor and motor recovery of patients during and after rehabilitation. Such evaluations cannot be reliably performed using standard clinical scales only. Furthermore, the combination of the analysis from different domains provides a comprehensive and complementary evaluation of the patient, which may help to better understand the organization and rearrangement of the central nervous and musculoskeletal systems [30]. The integration of multiple information sources allows for the identification of specific causes underlying the impairment that could not be discerned with clinical scales [124], and the implementation of multi-parameter assessments can identify motor improvements that cannot be detected with clinical scales or measured from one domain only. In Belfatto et al., a set-up for upper-limb robot-assisted rehabilitation was tested with a multi-parameter evaluation, showing that clinical improvements of the patients could be related to neurophysiological and motor changes measured by instrumental assessments before and after therapy [130] and that the assessments might provide complementary information or, in some cases, not even be in full agreement. In this study, post-stroke patients did not improve their movement range of motion significantly, but they could perform smoother movements, improving their motor control. This finding was in part reflected in their muscle synergies that were only slightly modified after the therapies and in EEG assessments that showed an enhancement of the desynchronization (especially in the contralateral hemisphere), which may reflect a motor recovery. Thus, multi-domain approaches may allow us to identify finer improvements that cannot be detected by clinal scales only. Indeed, relating the brain activations detected with EEG to changes in muscle recruitments and smoother movements may help to distinguish between functional recovery and the adoption of suboptimal compensatory strategies. Kinematic and kinetic measures are one of the most used and valuable methods used to assess motor performance, but they cannot detect compensatory strategies related to neural deficits if they are not related to electrophysiological measures [159]. In this way, multi-domain approaches provide a deeper insight into the mechanisms underlying the relearning procedure and the level (neuro/muscular) at which it occurred.

Each tool, device, or sensor provides specific information related to its specific domain, allowing for a deep—but limited to the domain—characterization of the patient. A more comprehensive characterization of the patient is needed to tailor rehabilitation therapies, since pathologies affect each patient differently [160], and multi-domain approaches allow us to evaluate neuromotor organization at different levels, facilitating the customization of the therapy in the patient [5]. In some studies, multi-parameter assessments could be employed successfully when associated with robotic rehabilitation. These approaches allow for measurements of biomechanical parameters with sensors directly integrated into the robotic tool, such as kinematic and force data, improving the objectivity, repeatability, precision, and easiness-to-use [161]. In this way, rehabilitation can be adapted rapidly based on the motor performance, which is computed easily and promptly by the set of integrated sensors [162].

It is indeed clear from available studies that multi-parameter approaches offer a set of interpretative advantages that may strongly affect the comprehension of the mechanisms underlying pathologies and clinical decision-making. It is, however, also clear that the field of multi-domain approaches is based on pilot studies and has not yet found wide application in clinical studies, since these approaches also show disadvantages.

### 4.2. Disadvantages of Multi-Domain Approaches

Multi-domain instrumental approaches have some limitations that have prevented their systematic use in clinical practice. First of all, employing multiple sensors would require a long preparation time that is not feasible for routine assessments in clinics, in which the time dedicated to a single patient is limited [163]. Moreover, all the equipment might be very expensive for facilities that have to invest in these technologies [164].

Furthermore, the use of simultaneous multiple recordings results in a large number of sensors and cables being attached to the patient. This leads to multiple possible consequences. First, from a technical point of view, sensors may interfere and cause relative movements or the detachment of the sensors themselves from the skin. Indeed, recordings can be contaminated by electronic equipment, such as power line noise and cable motion artifacts, and by movement artifacts at the electrode–skin interface [165]. Second, the discomfort of the patient could increase, affecting the psychological state and performance during the assessment, and the set of devices may lack transparency altering motion performance.

Another difficulty for clinical use is the effort required from the clinical personnel: clinicians need practice and training to use different equipment and for interpreting the results, or specific professional figures have to be employed [166,167]. This can be complex because of the often-unclear relationships between multimodal assessments (e.g., clinical and kinematic) and the high number of resources required to apply a battery of these assessments. Clinicians require evidence on the reliability of the different measures and the standardization for the use of assessment tasks and measurement systems, which are currently missing [168]. To spread the use of multi-domain approaches in clinical routine, it is necessary to define standardized protocols that guarantee the repeatability of measures.

### 4.3. Approaches to the Analysis of Multiple Domains in Clinical Practice

Most of the scientific articles presenting multi-domain approaches preliminary present studies in which physiological and pathological processes are investigated only in a limited number of patients or even in healthy controls. This set of pilot studies is needed to explore biomarkers that could potentially support diagnosis and decisions on therapy. However, at the present stage, few studies have employed multi-domain measures to evaluate the effects of rehabilitation on motor recovery during training sessions or in pre-post comparisons and longitudinal studies.

Even if the potential of multi-domain assessments has only been partially exploited, different approaches could be used for the integration of the parameters from multiple domains. The analytical approach is to analyze the domains separately to characterize each level of the neuro-muscle-skeletal system and, successively, compare the results to find if they are in accordance. In this way, each domain is fully characterized and all the information of each assessment can be captured, as in Belfatto et al. [130]; however, this approach can also lead to non-agreements. Another approach is to integrate the data from the different domains to find relations with data-driven methods. This approach was employed by Pierella et al., in which kinematic, EMG, and EEG signals were combined using principal components analysis (PCA) [131]. Similar approaches are the MMF algorithm that allows for extracting both muscular and kinematic synergies together and linking the muscle and task spaces in the same factorization [125], and the CMC, which searches for relations between the brain motor cortex and the associated muscles [112]. Analyzing the integrated domains allows us to find their correlations, detailing how the data agree; specific information of each domain may not be caught with the same level of detail as in the analysis of every single domain, as the information is “fused” in a synthetic approach.

### 4.4. Clinical Adoption of Multi-Domain Approach: Future Perspective and Barriers

There is a growing body of evidence that rehabilitation-focused biomarkers may provide more accurate information on patients’ stratification and help us to identify the proper respondent during different therapies. In this context, rehabilomics is a new emerging transdisciplinary science based on the evaluation of biomarkers, aiming to understand the rehabilitation-relevant phenotypes related to the biology, function, prognosis, treatment, and recovery of patients with neurological disabilities [169].

The concept behind this approach is that a given biological or molecular system can be better studied by considering it in its entirety, rather than in its individual elements. This principle is also at the basis of systems biology, according to which the study of an organism is considered an integrated and dynamic network of genes and proteins that interact with each other in space and time, allowing a specific system to function.

In this context, proteomics, genomics, and metabolomics, alongside functional brain imaging and neurophysiological technologies (i.e., MEPs, EEG, surface EMG, NIRS, and kinematics), have been explored as potential biomarkers of motor recovery in technology-assisted rehabilitation.

In clinical rehabilitation practice, the use of a rehabilomic approach along with multi-sensors assessment can be used, for example, to distinguish subjects who have a high risk of developing complications following the proposed rehabilitation treatment from subjects who will not manifest them precisely because of their genetic characteristics and their biomolecular profile. The use of novel biomarkers in the rehabilitation field is useful at several levels: in the preclinical phase, allowing us to identify from the genetic profile the subjects at risk of developing diseases of rehabilitative interest; when the disease has manifested itself, to tailor a treatment to the individual patient, improving the effectiveness and efficiency of the rehabilitation proposal; and finally, it is useful to monitor the effects of the proposal on the individual patient.

### 4.5. Future Research and Practice

To spread the use of multi-domain approaches for future applications, limitations of their use in clinical practice should be overcome. Summarizing the findings of our review, we identified specific future directions and barriers to large-scale adoption.

We expect that future research and practice should:

(i) Adopt standardized methods and criteria for selecting, combining, and analyzing multi-domain data to ensure the validity, reliability, and comparability of results across studies and settings [168]. To achieve this, it is necessary to match the study design with clinical needs in terms of setup complexity and time for preparation [163], and to define guidelines of good practice for all the combinations of assessments;
(ii) Validate multi-domain approaches against gold-standard measures and clinical outcomes to establish their accuracy, sensitivity, and specificity for different patient populations and conditions;
(iii) Integrate multi-domain approaches into clinical practice and research by developing user-friendly interfaces, protocols, and guidelines that facilitate their application and interpretation by clinicians and researchers. The comfort and psychological state of the patient has to be preserved, since the use of many sensors may interfere with the execution of tasks; thus, the sensors should be made less invasive. To foster the use of multi-domain approaches, instrumental measures and clinical observation must be linked so that clinicians may be more encouraged to apply multi-domain approaches and employ such techniques for evaluations and clinical decision-making. Clinicians or other professional figures have to be instructed on the use of the instrumentation and trained to interpret the results [167];
(iv) Explore novel multi-domain combinations and methods that can capture more aspects of motor function and recovery, such as neural plasticity, muscle metabolism, or cognitive–motor interactions.

### 4.6. Messages Learnt

To further clarify the main needs and open points, associated lessons learnt and solutions, we summarized the main points of discussion on the use of multi-domain approaches and the corresponding possible solutions in Table 1.

### 4.7. Limitations

This work has some limitations. First, the scope of the review is comprehensive and could not systematically cover all the papers available in the literature. While the authors are confident that the vast majority of relevant papers have been screened and reviewed, it is possible that a few studies have been missed. Due to the choice of a narrative approach, in this work no meta-analyses were presented and, thus, only the main messages were presented without detailed assessments. Considering that the field is still lacking clear evidence due to the frontier approach adopted by the inherent studies, our review could highlight many relevant points, but it is not yet able to set precise guidelines for clinical practice.

## 5. Conclusions

Multi-domain instrumental approaches showed great potential for improving clinical assessment and the rehabilitation of motor function by providing more comprehensive, objective, and personalized information on patients’ states and recovery. Despite some barriers to their systematic adoption in a clinical environment, they represent a fundamental step for research on neurophysiology and rehabilitation and a valuable option for standard clinical practice. Future research will contribute to the fostering of these approaches.

## Figures and Tables

**Table 1:** Summary of the main points of discussion on the use of multi-domain approaches and the corresponding possible solutions.

Needs and Open Points	Lessons Learnt and Solutions
Need for multi-domain assessments and approaches	The use of many different sensors allows us to characterize pathologies with a multifactorial approach, improving clinical standards
Provide homogeneous guidelines for data analysis	Adopt standardized methods and criteria for selecting, combining, and analyzing multi-domain data to ensure the validity, reliability, and comparability of results across studies and settings
Validate multi-domain approaches	Validate multi-domain approaches against gold-standard measures and clinical outcomes to establish their accuracy, sensitivity, and specificity for different patient populations and conditions
Some approaches do not show coherent outcomes	- They can highlight different aspects of the rehabilitation course, providing a more tridimensional assessment of patient status - They can help in spotting interpretation errors on specific domains
Preliminary recommendation for clinical practice	Explore novel multi-domain combinations and methods that can capture more aspects of motor function and recovery
Adopt multi-domain approaches as a clinical standard	Integrate multi-domain approaches into clinical practice and research by developing user-friendly interfaces, protocols, and guidelines that facilitate their application and interpretation by clinicians and researchers
Guarantee tolerable treatments and protocols to patients	Reduce the encumbrance and increase the transparency of multisensory approaches to improve patients’ tolerability
Conform multi-domain approaches to clinical time requirements	Reduce research protocols to their essence to be compliant to clinical timings

## Data Availability

Not applicable.

## Abbreviations

ALS: amyotrophic lateral sclerosis; ARAT: Action Research Arm Test; BCI: brain-computer interface; CHF: chronic heart failure; CMC: cortico-muscular coherence; CNS: central nervous systems; CP: cerebral palsy; CW: continuous wave; DCS: diffuse correlation spectroscopy; EC: effective connectivity; EEG: electroencephalography; EMG: electromyography; ERP: event-related potential; FC: Functional connectivity; fMRI: functional magnetic resonance imaging; FMT: Fugl–Meyer Test; fNIRS: functional near-infrared spectroscopy; ICF: International Classification of Functioning, Disability, and Health; ICU: intensive care unit; MEP: motor-evoked potential; MF: median frequency; MMF: mixed-matrix factorization; MPF: mean power frequency; MU: motor unit; MUAP: motor-unit action potential; NIRS: near-infrared spectroscopy; PCA: principal components analysis; PD: Parkinson’s disease; ROM: range of motion; RS: resting-state; SCI: spinal cord injury; TBI: traumatic brain injury; TD: time-domain; TMS: transcranial magnetic stimulation; WMFT: Wolf Motor Function Test.

## References

[1] Santisteban L., Térémetz M., Bleton J.P., Baron J.C., Maier M.A., Lindberg P.G. (2016). Upper limb outcome measures used in stroke rehabilitation studies: A systematic literature review. PLoS ONE.

[2] Skorvanek M., Goldman J.G., Jahanshahi M., Marras C., Rektorova I., Schmand B., van Duijn E., Goetz C.G., Weintraub D., Stebbins G.T. (2018). Global scales for cognitive screening in Parkinson’s disease: Critique and recommendations. Mov. Disord..

[3] MacEira-Elvira P., Popa T., Schmid A.C., Hummel F.C. (2019). Wearable technology in stroke rehabilitation: Towards improved diagnosis and treatment of upper-limb motor impairment. J. Neuroeng. Rehabil..

[4] Lambercy O., Maggioni S., Lünenburger L., Gassert R., Bolliger M. (2016). Robotic and wearable sensor technologies for measurements/clinical assessments. Neurorehabilitation Technology.

[5] Fleury L., Koch P.J., Wessel M.J., Bonvin C., San Millan D., Constantin C., Vuadens P., Adolphsen J., Cadic Melchior A., Brügger J. (2022). Toward individualized medicine in stroke-The TiMeS project: Protocol of longitudinal, multi-modal, multi-domain study in stroke. Front. Neurol..

[6] Levin M.F., Hiengkaew V., Nilanont Y., Cheung D., Dai D., Shaw J., Bayley M., Saposnik G. (2019). Relationship between Clinical Measures of Upper Limb Movement Quality and Activity Poststroke. Neurorehabil. Neural Repair.

[7] Gao Z., Pang Z., Chen Y., Lei G., Zhu S., Li G., Shen Y., Xu W. (2022). Restoring After Central Nervous System Injuries: Neural Mechanisms and Translational Applications of Motor Recovery. Neurosci. Bull..

[8] Alt Murphy M., Resteghini C., Feys P., Lamers I. (2015). An overview of systematic reviews on upper extremity outcome measures after stroke. BMC Neurol..

[9] Velstra I.M., Ballert C.S., Cieza A. (2011). A systematic literature review of outcome measures for upper extremity function using the international classification of functioning, disability, and health as reference. PM R.

[10] Timmermans A.A., Seelen H.A., Willmann R.D., Kingma H. (2009). Technology-assisted training of arm-hand skills in stroke: Concepts on reacquisition of motor control and therapist guidelines for rehabilitation technology design. J. Neuroeng. Rehabil..

[11] Lemmens R.J.M., Timmermans A.A.A., Janssen-Potten Y.J.M., Smeets R.J.E.M., Seelen H.A.M. (2012). Valid and reliable instruments for arm-hand assessment at ICF activity level in persons with hemiplegia: A systematic review. BMC Neurol..

[12] Lang C.E., Bland M.D., Bailey R.R., Schaefer S.Y., Birkenmeier R.L. (2013). Assessment of upper extremity impairment, function, and activity after stroke: Foundations for clinical decision making. J. Hand Ther..

[13] Fugl Meyer A.R., Jaasko L., Leyman I. (1975). The post stroke hemiplegic patient. I. A method for evaluation of physical performance. Scand. J. Rehabil. Med..

[14] Lyle R.C. (1981). A performance test for assessment of upper limb function in physical rehabilitation treatment and research. Int. J. Rehabil. Res..

[15] Wolf S.L., Lecraw D.E., Barton L.A., Jann B.B. (1989). Forced use of hemiplegic upper extremities to reverse the effect of learned nonuse among chronic stroke and head-injured patients. Exp. Neurol..

[16] Demers M., Levin M.F. (2017). Do Activity Level Outcome Measures Commonly Used in Neurological Practice Assess Upper-Limb Movement Quality?. Neurorehabil. Neural Repair.

[17] Gasperini G., Rota M., Guanziroli E., Bissolotti L., Balestrieri F., Chisari C., Currà A., Del Felice A., Farina N., Manganotti P. (2022). Development and Rasch Validation of an Observational Assessment Tool of Upper Limb Functional Impairment in Stroke Survivors: Functional Assessment Test for Upper Limb. Arch. Phys. Med. Rehabil..

[18] Kwakkel G., Lannin N.A., Borschmann K., English C., Ali M., Churilov L., Saposnik G., Winstein C., van Wegen E.E.H., Wolf S.L. (2017). Standardized measurement of sensorimotor recovery in stroke trials: Consensus-based core recommendations from the Stroke Recovery and Rehabilitation Roundtable. Int. J. Stroke.

[19] Veerbeek J.M., Kwakkel G., Van Wegen E.E.H., Ket J.C.F., Heymans M.W. (2011). Early prediction of outcome of activities of daily living after stroke: A systematic review. Stroke.

[20] Alt Murphy M., Al-Shallawi A., Sunnerhagen K.S., Pandyan A. (2022). Early prediction of upper limb functioning after stroke using clinical bedside assessments: A prospective longitudinal study. Sci. Rep..

[21] De Los Reyes-Guzmán A., Dimbwadyo-Terrer I., Trincado-Alonso F., Monasterio-Huelin F., Torricelli D., Gil-Agudo A. (2014). Quantitative assessment based on kinematic measures of functional impairments during upper extremity movements: A review. Clin. Biomech..

[22] Mazzoleni S., Do Tran V., Dario P., Posteraro F. (2018). Wrist Robot-Assisted Rehabilitation Treatment in Subacute and Chronic Stroke Patients: From Distal-to-Proximal Motor Recovery. IEEE Trans. Neural Syst. Rehabil. Eng..

[23] Van Dokkum L., Hauret I., Mottet D., Froger J., Métrot J., Laffont I. (2014). The contribution of kinematics in the assessment of upper limb motor recovery early after stroke. Neurorehabil. Neural Repair.

[24] Balasubramanian S., Melendez-Calderon A., Burdet E. (2012). A robust and sensitive metric for quantifying movement smoothness. IEEE Trans. Biomed. Eng..

[25] Lang C.E., Wagner J.M., Bastian A.J., Hu Q., Edwards D.F., Sahrmann S.A., Dromerick A.W. (2005). Deficits in grasp versus reach during acute hemiparesis. Exp. Brain Res..

[26] Hussain N., Alt Murphy M., Lundgren-Nilsson Å., Sunnerhagen K.S. (2020). Relationship between self-reported and objectively measured manual ability varies during the first year post-stroke. Sci. Rep..

[27] Rohrer B., Fasoli S., Krebs H.I., Hughes R., Volpe B., Frontera W.R., Stein J., Hogan N. (2002). Movement smoothness changes during stroke recovery. J. Neurosci..

[28] Palermo E., Hayes D.R., Russo E.F., Calabrò R.S., Pacilli A., Filoni S. (2018). Translational effects of robot-mediated therapy in subacute stroke patients: An experimental evaluation of upper limb motor recovery. PeerJ.

[29] Balasubramanian S., Melendez-Calderon A., Roby-Brami A., Burdet E. (2015). On the analysis of movement smoothness. J. Neuroeng. Rehabil..

[30] Maura R.M., Rueda Parra S., Stevens R.E., Weeks D.L., Wolbrecht E.T., Perry J.C. (2023). Literature review of stroke assessment for upper-extremity physical function via EEG, EMG, kinematic, and kinetic measurements and their reliability. J. Neuroeng. Rehabil..

[31] Drost G., Stegeman D.F., van Engelen B.G.M., Zwarts M.J. (2006). Clinical applications of high-density surface EMG: A systematic review. J. Electromyogr. Kinesiol..

[32] Farina D., Merletti R., Enoka R.M. (2014). The extraction of neural strategies from the surface EMG: An update. J. Appl. Physiol..

[33] Thornton R.C., Michell A.W. (2012). Techniques and applications of EMG: Measuring motor units from structure to function. J. Neurol..

[34] Benedetti M.G., Bonato P., Catani F., D’Alessio T., Knaflitz M., Marcacci M., Simoncini L. (1999). Myoelectric activation pattern during gait in total knee replacement: Relationship with kinematics, kinetics, and clinical outcome. IEEE Trans. Rehabil. Eng..

[35] Cheung V.C.K., D’Avella A., Bizzi E. (2009). Adjustments of motor pattern for load compensation via modulated activations of muscle synergies during natural behaviors. J. Neurophysiol..

[36] Bizzi E., Cheung V.C.K., D’Avella A., Saltiel P., Tresch M. (2008). Combining modules for movement. Brain Res. Rev..

[37] D’Avella A., Bizzi E. (2005). Shared and specific muscle synergies in natural motor behaviors. Proc. Natl. Acad. Sci. USA.

[38] Cifrek M., Medved V., Tonković S., Ostojić S. (2009). Surface EMG based muscle fatigue evaluation in biomechanics. Clin. Biomech..

[39] Wang L., Wang Y., Ma A., Ma G., Ye Y., Li R., Lu T. (2018). A Comparative Study of EMG Indices in Muscle Fatigue Evaluation Based on Grey Relational Analysis during All-Out Cycling Exercise. Biomed Res. Int..

[40] Al-Mulla M.R., Sepulveda F., Colley M. (2011). A review of non-invasive techniques to detect and predict localised muscle fatigue. Sensors.

[41] Farina D., Merletti R., Enoka R.M. (2004). The extraction of neural strategies from the surface EMG. J. Appl. Physiol..

[42] Stegeman D.F., Blok J.H., Hermens H.J., Roeleveld K. (2000). Surface EMG models: Properties and applications. J. Electromyogr. Kinesiol..

[43] Chowdhury R.H., Reaz M.B.I., Bin Mohd Ali M.A., Bakar A.A.A., Chellappan K., Chang T.G. (2013). Surface electromyography signal processing and classification techniques. Sensors.

[44] Campanini I., Disselhorst-Klug C., Rymer W.Z., Merletti R. (2020). Surface EMG in Clinical Assessment and Neurorehabilitation: Barriers Limiting Its Use. Front. Neurol..

[45] Patten C., Lexell J., Brown H.E. (2004). Weakness and strength training in persons with poststroke hemiplegia: Rationale, method, and efficacy. J. Rehabil. Res. Dev..

[46] MG B., DJ C., SA K. (2010). Evaluation of abnormal synergy patterns poststroke: Relationship of the Fugl-Meyer Assessment to hemiparetic locomotion. Neurorehabil. Neural Repair.

[47] Cheung V.C.K., Turolla A., Agostini M., Silvoni S., Bennis C., Kasi P., Paganoni S., Bonato P., Bizzi E. (2012). Muscle synergy patterns as physiological markers of motor cortical damage. Proc. Natl. Acad. Sci. USA.

[48] Kiguchi K., Hayashi Y. (2012). An EMG-based control for an upper-limb power-assist exoskeleton robot. IEEE Trans. Syst. Man, Cybern. Part B Cybern..

[49] Scheme E., Englehart K. (2011). Electromyogram pattern recognition for control of powered upper-limb prostheses: State of the art and challenges for clinical use. J. Rehabil. Res. Dev..

[50] McDonald A.C., Mulla D.M., Keir P.J. (2019). Using EMG Amplitude and Frequency to Calculate a Multimuscle Fatigue Score and Evaluate Global Shoulder Fatigue. Hum. Factors.

[51] Scano A., Re R., Tomba A., Amata O., Pirovano I., Brambilla C., Contini D., Spinelli L., Amendola C., Caserta A.V. (2023). Non-Surgical Lower-Limb Rehabilitation Enhances Quadriceps Strength in Inpatients with Hip Fracture : A Study on Force Capacity and Fatigue. Appl. Sci..

[52] Nunez P.L., Srinivasan R. (2009). Electric Fields of the Brain: The neurophysics of EEG.

[53] Teplan M. (2002). Fundamentals of EEG measurement. Meas. Sci. Rev..

[54] Seeber M., Cantonas L.M., Hoevels M., Sesia T., Visser-Vandewalle V., Michel C.M. (2019). Subcortical electrophysiological activity is detectable with high-density EEG source imaging. Nat. Commun..

[55] Friston K.J. (2011). Functional and Effective Connectivity: A Review. Brain Connect..

[56] Pereda E., Quiroga R.Q., Bhattacharya J. (2005). Nonlinear multivariate analysis of neurophysiological signals. Prog. Neurobiol..

[57] Blinowska K.J. (2011). Review of the methods of determination of directed connectivity from multichannel data. Med. Biol. Eng. Comput..

[58] Rubinov M., Sporns O. (2010). Complex network measures of brain connectivity: Uses and interpretations. Neuroimage.

[59] Stinear C.M. (2017). Prediction of motor recovery after stroke: Advances in biomarkers. Lancet. Neurol..

[60] Finnigan S.P., Walsh M., Rose S.E., Chalk J.B. (2007). Quantitative EEG indices of sub-acute ischaemic stroke correlate with clinical outcomes. Clin. Neurophysiol..

[61] Trujillo P., Mastropietro A., Scano A., Chiavenna A., Mrakic-Sposta S., Caimmi M., Molteni F., Rizzo G. (2017). Quantitative EEG for predicting upper limb motor recovery in chronic stroke robot-Assisted rehabilitation. IEEE Trans. Neural Syst. Rehabil. Eng..

[62] Wang Q., Meng L., Pang J., Zhu X., Ming D. (2020). Characterization of EEG Data Revealing Relationships with Cognitive and Motor Symptoms in Parkinson’s Disease: A Systematic Review. Front. Aging Neurosci..

[63] Reid L.B., Rose S.E., Boyd R.N. (2015). Rehabilitation and neuroplasticity in children with unilateral cerebral palsy. Nat. Rev. Neurol..

[64] Piazza C., Pirovano I., Mastropietro A., Genova C., Gagliardi C., Turconi A.C., Malerba G., Panzeri D., Maghini C., Reni G. (2021). Development and preliminary testing of a system for the multimodal analysis of gait training in a virtual reality environment. Electronics.

[65] Simis M., Doruk Camsari D., Imamura M., Filippo T.R.M., Rubio De Souza D., Battistella L.R., Fregni F. (2021). Electroencephalography as a Biomarker for Functional Recovery in Spinal Cord Injury Patients. Front. Hum. Neurosci..

[66] De Vico Fallani F., Sinatra R., Astolfi L., Mattia D., Cincotti F., Latora V., Salinari S., Marciani M.G., Colosimo A., Babiloni F. Community structure of cortical networks in spinal cord injured patients. Proceedings of the 30th Annual International Conference of the IEEE Engineering in Medicine and Biology Society, EMBS’08—“Personalized Healthcare through Technology”.

[67] Irimia A., Van Horn J.D. (2015). Functional neuroimaging of traumatic brain injury: Advances and clinical utility. Neuropsychiatr. Dis. Treat..

[68] Bistriceanu C.E., Danciu F.A., Cuciureanu D.I. (2022). Cortical connectivity in stroke using signals from resting-state EEG: A review of current literature. Acta Neurol. Belg..

[69] Hordacre B., Goldsworthy M.R., Welsby E., Graetz L., Ballinger S., Hillier S. (2020). Resting State Functional Connectivity Is Associated with Motor Pathway Integrity and Upper-Limb Behavior in Chronic Stroke. Neurorehabil. Neural Repair.

[70] De Vico Fallani F., Clausi S., Leggio M., Chavez M., Valencia M., Maglione A.G., Babiloni F., Cincotti F., Mattia D., Molinari M. (2017). Interhemispheric Connectivity Characterizes Cortical Reorganization in Motor-Related Networks After Cerebellar Lesions. Cerebellum.

[71] Hoshino T., Oguchi K., Inoue K., Hoshino A., Hoshiyama M. (2021). Relationship between lower limb function and functional connectivity assessed by EEG among motor-related areas after stroke. Top. Stroke Rehabil..

[72] De Vico Fallani F., Astolfi L., Cincotti F., Mattia D., La Rocca D., Maksuti E., Salinari S., Babiloni F., Vegso B., Kozmann G. (2009). Evaluation of the brain network organization from EEG signals: A preliminary evidence in stroke patient. Anat. Rec..

[73] Wang X., Wong W.W., Sun R., Chu W.C.W., Tong K.Y. (2018). Differentiated effects of robot hand training with and without neural guidance on neuroplasticity patterns in chronic stroke. Front. Neurol..

[74] Samogin J., Marino M., Porcaro C., Wenderoth N., Dupont P., Swinnen S.P., Mantini D. (2020). Frequency-dependent functional connectivity in resting state networks. Hum. Brain Mapp..

[75] Romeo Z., Mantini D., Durgoni E., Passarini L., Meneghello F., Zorzi M. (2021). Electrophysiological signatures of resting state networks predict cognitive deficits in stroke. Cortex.

[76] Caliandro P., Vecchio F., Miraglia F., Reale G., Della Marca G., La Torre G., Lacidogna G., Iacovelli C., Padua L., Bramanti P. (2017). Small-World Characteristics of Cortical Connectivity Changes in Acute Stroke. Neurorehabil. Neural Repair.

[77] Vecchio F., Caliandro P., Reale G., Miraglia F., Piludu F., Masi G., Iacovelli C., Simbolotti C., Padua L., Leone E. (2019). Acute cerebellar stroke and middle cerebral artery stroke exert distinctive modifications on functional cortical connectivity: A comparative study via EEG graph theory. Clin. Neurophysiol..

[78] Vecchio F., Tomino C., Miraglia F., Iodice F., Erra C., Di Iorio R., Judica E., Alù F., Fini M., Rossini P.M. (2019). Cortical connectivity from EEG data in acute stroke: A study via graph theory as a potential biomarker for functional recovery. Int. J. Psychophysiol..

[79] Nicolo P., Magnin C., Pedrazzini E., Plomp G., Mottaz A., Schnider A., Guggisberg A.G. (2018). Comparison of Neuroplastic Responses to Cathodal Transcranial Direct Current Stimulation and Continuous Theta Burst Stimulation in Subacute Stroke. Arch. Phys. Med. Rehabil..

[80] Molteni F., Formaggio E., Bosco A., Guanziroli E., Piccione F., Masiero S., Del Felice A. (2020). Brain Connectivity Modulation after Exoskeleton-Assisted Gait in Chronic Hemiplegic Stroke Survivors: A Pilot Study. Am. J. Phys. Med. Rehabil..

[81] Maggio M.G., Naro A., Manuli A., Maresca G., Balletta T., Latella D., De Luca R., Calabrò R.S. (2021). Effects of Robotic Neurorehabilitation on Body Representation in Individuals with Stroke: A Preliminary Study Focusing on an EEG-Based Approach. Brain Topogr..

[82] Pichiorri F., Petti M., Caschera S., Astolfi L., Cincotti F., Mattia D. (2018). An EEG index of sensorimotor interhemispheric coupling after unilateral stroke: Clinical and neurophysiological study. Eur. J. Neurosci..

[83] Fanciullacci C., Panarese A., Spina V., Lassi M., Mazzoni A., Artoni F., Micera S., Chisari C. (2021). Connectivity Measures Differentiate Cortical and Subcortical Sub-Acute Ischemic Stroke Patients. Front. Hum. Neurosci..

[84] Park C.H., Chang W.H., Ohn S.H., Kim S.T., Bang O.Y., Pascual-Leone A., Kim Y.H. (2011). Longitudinal changes of resting-state functional connectivity during motor recovery after stroke. Stroke.

[85] Pirovano I., Mastropietro A., Antonacci Y., Barà C., Guanziroli E., Molteni F., Faes L., Rizzo G. (2022). Resting State EEG Directed Functional Connectivity Unveils Changes in Motor Network Organization in Subacute Stroke Patients After Rehabilitation. Front. Physiol..

[86] Wu J., Quinlan E.B., Dodakian L., McKenzie A., Kathuria N., Zhou R.J., Augsburger R., See J., Le V.H., Srinivasan R. (2015). Connectivity measures are robust biomarkers of cortical function and plasticity after stroke. Brain.

[87] Philips G.R., Daly J.J., Príncipe J.C. (2017). Topographical measures of functional connectivity as biomarkers for post-stroke motor recovery. J. Neuroeng. Rehabil..

[88] Asadi B., Cuenca-Zaldivar J.N., Nakhostin Ansari N., Ibáñez J., Herrero P., Calvo S. (2023). Brain Analysis with a Complex Network Approach in Stroke Patients Based on Electroencephalography: A Systematic Review and Meta-Analysis. Healthcare.

[89] Quaresima V., Ferrari M. (2019). A Mini-Review on Functional Near-Infrared Spectroscopy (fNIRS): Where Do We Stand, and Where Should We Go?. Photonics.

[90] Grassi B., Quaresima V. (2016). Near-infrared spectroscopy and skeletal muscle oxidative function in vivo in health and disease: A review from an exercise physiology perspective. J. Biomed. Opt..

[91] Scholkmann F., Kleiser S., Metz A.J., Zimmermann R., Mata Pavia J., Wolf U., Wolf M. (2014). A review on continuous wave functional near-infrared spectroscopy and imaging instrumentation and methodology. Neuroimage.

[92] Torricelli A., Contini D., Pifferi A., Caffini M., Re R., Zucchelli L., Spinelli L. (2014). Time domain functional NIRS imaging for human brain mapping. Neuroimage.

[93] Durduran T., Yodh A.G. (2014). Diffuse correlation spectroscopy for non-invasive, micro-vascular cerebral blood flow measurement. Neuroimage.

[94] Giacalone G., Zanoletti M., Re R., Germinario B., Contini D., Spinelli L., Torricelli A., Roveri L. (2019). Time-domain near-infrared spectroscopy in acute ischemic stroke patients. Neurophotonics.

[95] Roldán M., Kyriacou P.A. (2021). Near-infrared spectroscopy (NIRS) in traumatic brain injury (TBI). Sensors.

[96] Srinivasan K., Currim F., Lindberg C.M., Razjouyan J., Gilligan B., Lee H., Canada K.J., Goebel N., Mehl M.R., Lunden M.M. (2023). Discovery of associative patterns between workplace sound level and physiological wellbeing using wearable devices and empirical Bayes modeling. npj Digit. Med..

[97] Thewissen L., Caicedo A., Lemmers P., Van Bel F.V., Van Huffel S.V., Naulaers G. (2018). Measuring near-infrared spectroscopy derived cerebral autoregulation in neonates: From research tool toward bedside multimodal monitoring. Front. Pediatr..

[98] Mihara M., Miyai I. (2016). Review of functional near-infrared spectroscopy in neurorehabilitation. Neurophotonics.

[99] Lin J.H., Maikala R.V., McGorry R., Brunette C. (2010). NIRS application in evaluating threaded-fastener driving assembly tasks. Int. J. Ind. Ergon..

[100] Bonnal J., Monnet F., Le B.T., Pila O., Grosmaire A.G., Ozsancak C., Duret C., Auzou P. (2022). Relation between Cortical Activation and Effort during Robot-Mediated Walking in Healthy People: A Functional Near-Infrared Spectroscopy Neuroimaging Study (fNIRS). Sensors.

[101] Lacerenza M., Spinelli L., Buttafava M., Dalla Mora A., Zappa F., Pifferi A., Tosi A., Cozzi B., Torricelli A., Contini D. (2021). Monitoring the motor cortex hemodynamic response function in freely moving walking subjects: A time-domain fNIRS pilot study. Neurophotonics.

[102] Xie F., Huang S., Miao T., He S., Lin Z., Xie L. (2022). Development of a Wireless Multichannel Near-Infrared Spectroscopy Sensor System for Monitoring Muscle Activity. IEEE Sens. J..

[103] Scholkmann F., Tachtsidis I., Wolf M., Wolf U. (2022). Systemic physiology augmented functional near-infrared spectroscopy: A powerful approach to study the embodied human brain. Neurophotonics.

[104] Barstow T.J. (2019). Understanding near infrared spectroscopy and its application to skeletal muscle research. J. Appl. Physiol..

[105] Tuesta M., Yáñez-Sepúlveda R., Verdugo-Marchese H., Mateluna C., Alvear-Ordenes I. (2022). Near-Infrared Spectroscopy Used to Assess Physiological Muscle Adaptations in Exercise Clinical Trials: A Systematic Review. Biology.

[106] Adami A., Rossiter H.B. (2018). Principles, insights, and potential pitfalls of the noninvasive determination of muscle oxidative capacity by near-infrared spectroscopy. J. Appl. Physiol..

[107] Wȩgrzynowska-Teodorczyk K., Siennicka A., Josiak K., Zymliński R., Kasztura M., Banasiak W., Ponikowski P., Woźniewski M. (2018). Evaluation of Skeletal Muscle Function and Effects of Early Rehabilitation during Acute Heart Failure: Rationale and Study Design. Biomed Res. Int..

[108] Manfredini F., Lamberti N., Ficarra V., Tsolaki E., Straudi S., Zamboni P., Basaglia N., Gasbarro V. (2020). Biomarkers of muscle metabolism in peripheral artery disease: A dynamic NIRS-assisted study to detect adaptations following revascularization and exercise training. Diagnostics.

[109] Soares R.N., Murias J.M., Saccone F., Puga L., Moreno G., Resnik M., De Roia G.F. (2019). Effects of a rehabilitation program on microvascular function of CHD patients assessed by near-infrared spectroscopy. Physiol. Rep..

[110] Ferrante J.M., Piasecki A.K., Ohman-Strickland P.A., Crabtree B.F. (2009). Family physicians’ practices and attitudes regarding care of extremely obese patients. Obesity.

[111] Brambilla C., Pirovano I., Mira R.M., Rizzo G., Scano A., Mastropietro A. (2021). Combined use of emg and eeg techniques for neuromotor assessment in rehabilitative applications: A systematic review. Sensors.

[112] Liu J., Sheng Y., Liu H. (2019). Corticomuscular coherence and its applications: A review. Front. Hum. Neurosci..

[113] von Carlowitz-Ghori K., Bayraktaroglu Z., Hohlefeld F.U., Losch F., Curio G., Nikulin V.V. (2014). Corticomuscular coherence in acute and chronic stroke. Clin. Neurophysiol..

[114] Pan L.L.H., Yang W.W., Kao C.L., Tsai M.W., Wei S.H., Fregni F., Chen V.C.F., Chou L.W. (2018). Effects of 8-week sensory electrical stimulation combined with motor training on EEG-EMG coherence and motor function in individuals with stroke. Sci. Rep..

[115] Calabrò R.S., Naro A., Russo M., Bramanti P., Carioti L., Balletta T., Buda A., Manuli A., Filoni S., Bramanti A. (2018). Shaping neuroplasticity by using powered exoskeletons in patients with stroke: A randomized clinical trial. J. Neuroeng. Rehabil..

[116] Major Z.Z., Vaida C., Major K.A., Tucan P., Simori G., Banica A., Brusturean E., Burz A., Craciunas R., Ulinici I. (2020). The impact of robotic rehabilitation on the motor system in neurological diseases. A multimodal neurophysiological approach. Int. J. Environ. Res. Public Health.

[117] Kim B., Kim L., Kim Y.H., Yoo S.K. (2017). Cross-association analysis of EEG and EMG signals according to movement intention state. Cogn. Syst. Res..

[118] Bao S.C., Wong W.W., Leung T.W.H., Tong K.Y. (2019). Cortico-Muscular Coherence Modulated by High-Definition Transcranial Direct Current Stimulation in People with Chronic Stroke. IEEE Trans. Neural Syst. Rehabil. Eng..

[119] Yang H., Wan J., Jin Y., Yu X., Fang Y. (2022). EEG- and EMG-Driven Poststroke Rehabilitation: A Review. IEEE Sens. J..

[120] D’Addio G., Cesarelli M., Romano M., Faiella G., Lullo F., Pappone N. Kinematic and EMG patterns evaluation of upper arm reaching movements. Proceedings of the IEEE RAS and EMBS International Conference on Biomedical Robotics and Biomechatronics.

[121] Kim J., Kim H., Kim J. Quantitative assessment test for upper-limb motor function by using EMG and kinematic analysis in the practice of occupational therapy. Proceedings of the Annual International Conference of the IEEE Engineering in Medicine and Biology Society, EMBS.

[122] De Marchis C., Patané F., Petrarca M., Carniel S., Schmid M., Conforto S., Castelli E., Cappa P., D’Alessio T. (2014). EMG and kinematics assessment of postural responses during balance perturbation on a 3D robotic platform: Preliminary results in children with hemiplegia. XIII Mediterranean Conference on Medical and Biological Engineering and Computing.

[123] Zhang X., Tang X., Zhu X., Gao X., Chen X., Chen X. (2019). A regression-based framework for quantitative assessment of muscle spasticity using combined emg and inertial data from wearable sensors. Front. Neurosci..

[124] Campanini I., Cosma M., Manca M., Merlo A. (2020). Added Value of Dynamic EMG in the Assessment of the Equinus and the Equinovarus Foot Deviation in Stroke Patients and Barriers Limiting Its Usage. Front. Neurol..

[125] Scano A., Mira R.M., D’Avella A. (2022). Mixed matrix factorization: A novel algorithm for the extraction of kinematic-muscular synergies. J. Neurophysiol..

[126] Mazzoleni S., Coscia M., Rossi G., Aliboni S., Posteraro F., Carrozza M.C. Effects of an upper limb robot-mediated therapy on paretic upper limb in chronic hemiparetic subjects: A biomechanical and EEG-based approach for functional assessment. Proceedings of the 2009 IEEE International Conference on Rehabilitation Robotics, ICORR 2009.

[127] Comani S., Schinaia L., Tamburro G., Velluto L., Sorbi S., Conforto S., Guarnieri B. Assessing neuro-motor recovery in a stroke survivor with high-resolution EEG, robotics and Virtual Reality. Proceedings of the Annual International Conference of the IEEE Engineering in Medicine and Biology Society, EMBS.

[128] Molteni E., Preatoni E., Cimolin V., Bianchi A.M., Galli M., Rodano R. A methodological study for the multifactorial assessment of Motor Adaptation: Integration of kinematic and neural factors. Proceedings of the 2010 Annual International Conference of the IEEE Engineering in Medicine and Biology Society, EMBC’10.

[129] Caimmi M., Visani E., Digiacomo F., Scano A., Chiavenna A., Gramigna C., Molinari Tosatti L., Franceschetti S., Molteni F., Panzica F. (2016). Predicting Functional Recovery in Chronic Stroke Rehabilitation Using Event-Related Desynchronization-Synchronization during Robot-Assisted Movement. Biomed Res. Int..

[130] Belfatto A., Scano A., Chiavenna A., Mastropietro A., Mrakic-Sposta S., Pittaccio S., Tosatti L.M., Molteni F., Rizzo G. (2018). A multiparameter approach to evaluate post-stroke patients: An application on robotic rehabilitation. Appl. Sci..

[131] Pierella C., Pirondini E., Kinany N., Coscia M., Giang C., Miehlbradt J., Magnin C., Nicolo P., Dalise S., Sgherri G. (2020). A multimodal approach to capture post-stroke temporal dynamics of recovery. J. Neural Eng..

[132] Scano A., Zanoletti M., Pirovano I., Spinelli L., Contini D., Torricelli A., Re R. (2019). NIRS-EMG for clinical applications: A systematic review. Appl. Sci..

[133] Taelman J., Vanderhaegen J., Robijns M., Naulaers G., Spaepen A., Huffel S. (2011). Van Estimation of Muscle Fatigue Using Surface Electromyography and Near-Infrared Spectroscopy. Adv. Exp. Med. Biol..

[134] Scano A., Pirovano I., Manunza M.E., Spinelli L., Contini D., Torricelli A., Re R. (2020). Sustained fatigue assessment during isometric exercises with time-domain near infrared spectroscopy and surface electromyography signals. Biomed. Opt. Express.

[135] Re R., Scano A., Pirovano I., Manunza M.E., Spinelli L., Contini D., Torricelli A. (2021). Assessment of muscular sustained fatigue: A TD-NIRS and sEMG study. Proceedings of the Optics InfoBase Conference Papers.

[136] Kankaanpää M., Colier W.N., Taimela S., Anders C., Airaksinen O., Kokko-Aro S.M., Hänninen O. (2005). Back extensor muscle oxygenation and fatigability in healthy subjects and low back pain patients during dynamic back extension exertion. Pathophysiology.

[137] Søgaard K., Blangsted A.K., Nielsen P.K., Hansen L., Andersen L.L., Vedsted P., Sjøgaard G. (2012). Changed activation, oxygenation, and pain response of chronically painful muscles to repetitive work after training interventions: A randomized controlled trial. Eur. J. Appl. Physiol..

[138] Jigjid E., Kawashima N., Ogata H., Nakazawa K., Akai M., Eto F., Haga N. (2008). Effects of passive leg movement on the oxygenation level of lower limb muscle in chronic stroke patients. Neurorehabil. Neural Repair.

[139] Kawashima N., Nakazawa K., Akai M. (2005). Muscle oxygenation of the paralyzed lower limb in spinal cord-injured persons. Med. Sci. Sports Exerc..

[140] Murray D., Keyser R.E., Chin L.M.K., Bulea T.C., Wutzke C.J., Guccione A.A. (2022). EMG median frequency shifts without change in muscle oxygenation following novel locomotor training in individuals with incomplete spinal cord injury. Disabil. Rehabil..

[141] Chiarelli A.M., Zappasodi F., Di Pompeo F., Merla A. (2017). Simultaneous functional near-infrared spectroscopy and electroencephalography for monitoring of human brain activity and oxygenation: A review. Neurophotonics.

[142] Blanco-Mora D.A., Almeida Y., Vieira C., Badia S.B.I. (2019). A study on EEG power and connectivity in a virtual reality bimanual rehabilitation training system. Proceedings of the Conference Proceedings—IEEE International Conference on Systems, Man and Cybernetics.

[143] Li J., Thakor N., Bezerianos A. (2020). Brain Functional Connectivity in Unconstrained Walking with and without an Exoskeleton. IEEE Trans. Neural Syst. Rehabil. Eng..

[144] Abtahi M., Bahram Borgheai S., Jafari R., Constant N., Diouf R., Shahriari Y., Mankodiya K. (2020). Merging fNIRS-EEG Brain Monitoring and Body Motion Capture to Distinguish Parkinsons Disease. IEEE Trans. Neural Syst. Rehabil. Eng..

[145] Dutta A., Jacob A., Chowdhury S.R., Das A., Nitsche M.A. (2015). EEG-NIRS Based Assessment of Neurovascular Coupling During Anodal Transcranial Direct Current Stimulation—A Stroke Case Series. J. Med. Syst..

[146] Jindal U., Sood M., Dutta A., Chowdhury S.R. (2015). Development of point of care testing device for neurovascular coupling from simultaneous recording of EEG and NIRS during anodal transcranial direct current stimulation. IEEE J. Transl. Eng. Health Med..

[147] Othman M.H., Bhattacharya M., Møller K., Kjeldsen S., Grand J., Kjaergaard J., Dutta A., Kondziella D. (2021). Resting-State NIRS–EEG in Unresponsive Patients with Acute Brain Injury: A Proof-of-Concept Study. Neurocrit. Care.

[148] Berger A., Horst F., Müller S., Steinberg F., Doppelmayr M. (2019). Current state and future prospects of EEG and fNIRS in robot-assisted gait rehabilitation: A brief review. Front. Hum. Neurosci..

[149] Wang Z., Cao C., Chen L., Gu B., Liu S., Xu M., He F., Ming D. (2022). Multimodal Neural Response and Effect Assessment During a BCI-Based Neurofeedback Training After Stroke. Front. Neurosci..

[150] Durduran T., Zhou C., Buckley E.M., Kim M.N., Yu G., Choe R., Gaynor J.W., Spray T.L., Durning S.M., Mason S.E. (2010). Optical measurement of cerebral hemodynamics and oxygen metabolism in neonates with congenital heart defects. J. Biomed. Opt..

[151] Rajaram A., Yip L.C.M., Milej D., Suwalski M., Kewin M., Lo M., Carson J.J.L., Han V., Bhattacharya S., Diop M. (2020). Perfusion and metabolic neuromonitoring during ventricular taps in infants with post-hemorrhagic ventricular dilatation. Brain Sci..

[152] De Carli A., Andresen B., Giovannella M., Durduran T., Contini D., Spinelli L., Weigel U.M., Passera S., Pesenti N., Mosca F. (2019). Cerebral oxygenation and blood flow in term infants during postnatal transition: BabyLux project. Arch. Dis. Child. Fetal Neonatal Ed..

[153] Guoqiang Yu K.G. (2013). Diffuse Correlation Spectroscopy (DCS) for Assessment of Tissue Blood Flow in Skeletal Muscle: Recent Progress. Anat. Physiol..

[154] Zanoletti M., Amendola C., Buttafava M., Carteano T., Contini D., Cortese L., Demarteau L., Frabasile L., Sagarzazu E.G., Guadagno C.N. (2022). VASCOVID: Hybrid diffuse optical platform combined with a pulse-oximeter and an automatized inflatable tourniquet for the assessment of metabolism and endothelial health in the intensive care. Proceedings of the Optics InfoBase Conference Papers.

[155] Baker W.B., Li Z., Schenkel S.S., Chandra M., Busch D.R., Englund E.K., Schmitz K.H., Yodh A.G., Floyd T.F., Mohler E.R. (2017). Effects of exercise training on calf muscle oxygen extraction and blood flow in patients with peripheral artery disease. J. Appl. Physiol..

[156] Quaresima V., Farzam P., Anderson P., Farzam P.Y., Wiese D., Carp S.A., Ferrari M., Franceschini M.A. (2019). Diffuse correlation spectroscopy and frequency-domain near-infrared spectroscopy for measuring microvascular blood flow in dynamically exercising human muscles. J. Appl. Physiol..

[157] Watanabe T., Murase N., Kime R., Kurosawa Y., Fuse S., Hamaoka T. (2021). Effects of Exercise Training on Cardiac and Skeletal Muscle Functions in Patients with Chronic Heart Failure. Adv. Exp. Med. Biol..

[158] Whyte E., Thomas S., Marzolini S. (2022). Muscle Oxygenation of the Paretic and Nonparetic Legs During and After Arterial Occlusion in Chronic Stroke. J. Stroke Cerebrovasc. Dis..

[159] Buma F.E., van Kordelaar J., Raemaekers M., van Wegen E.E.H., Ramsey N.F., Kwakkel G. (2016). Brain activation is related to smoothness of upper limb movements after stroke. Exp. Brain Res..

[160] Gracies J.M., Pradines M., Ghédira M., Loche C.M., Mardale V., Hennegrave C., Gault-Colas C., Audureau E., Hutin E., Baude M. (2019). Guided Self-rehabilitation Contract vs conventional therapy in chronic stroke-induced hemiparesis: NEURORESTORE, a multicenter randomized controlled trial. BMC Neurol..

[161] Zollo L., Rossini L., Bravi M., Magrone G., Sterzi S., Guglielmelli E. (2011). Quantitative evaluation of upper-limb motor control in robot-aided rehabilitation. Med. Biol. Eng. Comput..

[162] Do Tran V., Dario P., Mazzoleni S. (2018). Kinematic measures for upper limb robot-assisted therapy following stroke and correlations with clinical outcome measures: A review. Med. Eng. Phys..

[163] Lang C.E., Barth J., Holleran C.L., Konrad J.D., Bland M.D. (2020). Implementation of wearable sensing technology for movement: Pushing forward into the routine physical rehabilitation care field. Sensors.

[164] Feldner H.A., Howell D., Kelly V.E., McCoy S.W., Steele K.M. (2019). “Look, Your Muscles Are Firing!”: A Qualitative Study of Clinician Perspectives on the Use of Surface Electromyography in Neurorehabilitation. Arch. Phys. Med. Rehabil..

[165] De Luca C.J., Donald Gilmore L., Kuznetsov M., Roy S.H. (2010). Filtering the surface EMG signal: Movement artifact and baseline noise contamination. J. Biomech..

[166] Manca A., Cereatti A., Bar-On L., Botter A., Della Croce U., Knaflitz M., Maffiuletti N.A., Mazzoli D., Merlo A., Roatta S. (2020). A Survey on the Use and Barriers of Surface Electromyography in Neurorehabilitation. Front. Neurol..

[167] Merletti R., Temporiti F., Gatti R., Gupta S., Sandrini G., Serrao M. (2023). Translation of surface electromyography to clinical and motor rehabilitation applications: The need for new clinical figures. Transl. Neurosci..

[168] Schwarz A., Kanzler C.M., Lambercy O., Luft A.R., Veerbeek J.M. (2019). Systematic review on kinematic assessments of upper limb movements after stroke. Stroke.

[169] Wagner A.K. (2010). TBI translational rehabilitation research in the 21st Century: Exploring a Rehabilomics research model. Eur. J. Phys. Rehabil. Med..

